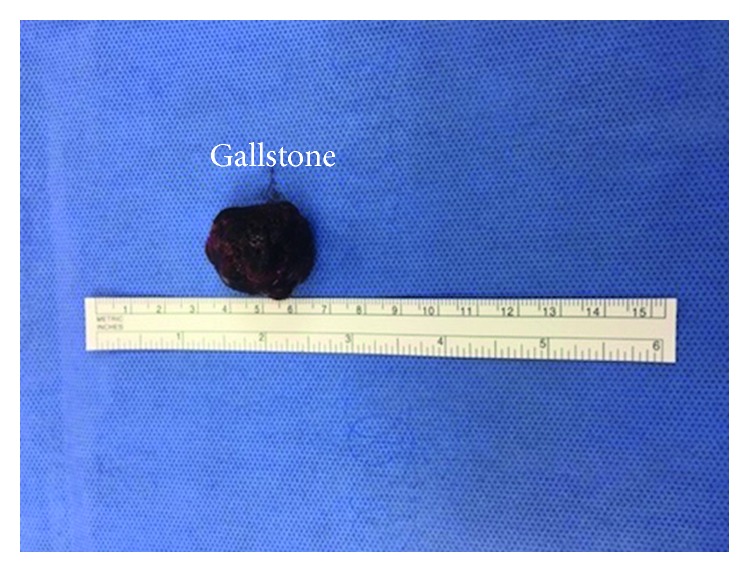# Free Intraperitoneal Gallstone: An Unusual Case of Small Bowel Obstruction from Extrinsic Compression

**DOI:** 10.1155/2018/1341572

**Published:** 2018-01-08

**Authors:** Kor Woi Tiang, Hang Fai So, Yang Hwang, Manjunath Siddaiah-Subramanya

**Affiliations:** ^1^Department of Surgery, Logan Hospital, Meadowbrook, QLD, Australia; ^2^Griffith University, Brisbane, QLD, Australia; ^3^University of Queensland, Brisbane, QLD, Australia

## Abstract

Laparoscopic cholecystectomy (LC) is preferred in the treatment of symptomatic cholecystolithiasis. Gallstone spillage is not uncommon, and there have been reports of associated complications. We report a case of a free intraperitoneal gallstone, left inadvertently during LC, which developed an inflammatory phlegmon with abscess containing gallstone, causing extraluminal compression on the distal ileum, resulting in small bowel obstruction. This complication in particular is almost unheard of. The patient underwent laparoscopic drainage of abscess and retrieval of gallstone, which relieved the obstruction. Clinicians, therefore, need to keep an open mind in the workup for bowel obstruction. During LC, gallstone spillage should be prevented and retrieved whenever possible to minimize early and late complications associated with it.

## 1. Introduction

Laparoscopic cholecystectomy (LC) is the gold standard for treating symptomatic gallstones, with large studies and systematic reviews reporting reduced hospital stay and pain [1, 2]. Complications from LC are relatively rare [3, 4]. Since the inception of LC in 1987 in France, the rate of unretrieved gallstones has been reported to be ranging between 1 and 40% [5–7]. Unretrieved gallstones commonly do not cause complications; however, recent literature suggests a range of early and late complications. These may include simple abscesses, fistula, and, rarely, small bowel obstruction [8–10]. This would suggest that unretrieved gallstones during LC should be recovered whenever possible. We present a rare case of small bowel obstruction from extrinsic compression secondary to one such unretrieved gallstone. In addition, we briefly discuss the consequences of unretrieved gallstones.

## 2. Case Report

An 83-year-old man presented to the emergency department (ED) with upper abdominal chest pain with newly deranged liver function tests (LFTs) and raised inflammatory markers. On examination, he was found to be tender in the right upper quadrant as well as in the epigastrium. His LFTs were as follows: unconjugated and conjugated bilirubin were both 26 and 14 *µ*mol/L, respectively; ALP, GGT, ALT, and AST were 333, 557, 194, and 158 U/L, respectively. His white cell count was 13.9 × 10^9^/L, and CRP was 105 mg/L. A computed tomography (CT) scan was performed due to his atypical presentation to the emergency department, which showed cholecystitis with a distended gallbladder containing a 21 mm solitary lamellated calcified calculus. This was then confirmed on abdominal ultrasound, which, in addition to the above findings, showed that there was no evidence of choledocholithiasis or biliary duct dilatation.

The patient underwent LC, performed by a senior surgical trainee with an intraoperative cholangiogram showing no filling defects in the biliary ducts and free flow of contrast into the duodenum. During the operation, the gallbladder was inadvertently opened whilst dissecting it from the liver bed, resulting in spillage of the large calculus. While retrieving the gallbladder specimen in an endoscopic bag, the spilled calculus was left inadvertently in the peritoneal cavity. The operator did not realize that the spilled stone was unretrieved. The patient made an uneventful, gradual recovery and was discharged on postoperative day 1. Macroscopic histopathology report revealed a partially opened gallbladder with no gallstone within the lumen, suggesting that the spilled large calculus was not retrieved from the peritoneal cavity. Microscopically, it was consistent with acute and chronic cholecystitis with muscle hypertrophy and fibrosis of the wall.

**Figure 1:**
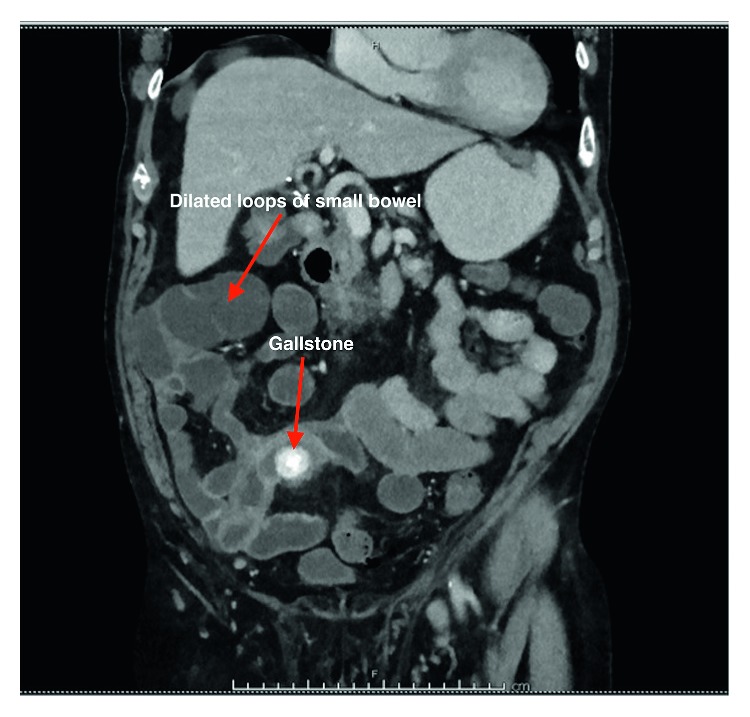


The patient re-presented to the emergency department 3 weeks after discharge with central abdominal pain, anorexia, and vomiting. He had been experiencing these symptoms since day 3 of discharge, and they had been getting worse until the time he re-presented. In spite of these symptoms, he was systemically well. Examination of the abdomen revealed minimally distended abdomen with tenderness in the periumbilical region. These symptoms and signs suggested that he had small bowel obstruction. However, in the setting of recent cholecystectomy, we wanted to rule out the possibility of a biliary injury. His LFTs and inflammatory markers were normal. His symptoms were further evaluated with a CT scan, which showed evidence of SBO with transition point in the right iliac fossa. The obstruction was from a laminated calcified structure applying extrinsic compression on a loop of small bowel. With the knowledge of the index operation and the pathology report of the retrieved specimen, it was suspected that this structure was the unretrieved gallstone. This calculus was associated with a surrounding phlegmon, evidenced by intense inflammatory change on the CT scan (Figure 1).

**Figure 2:**
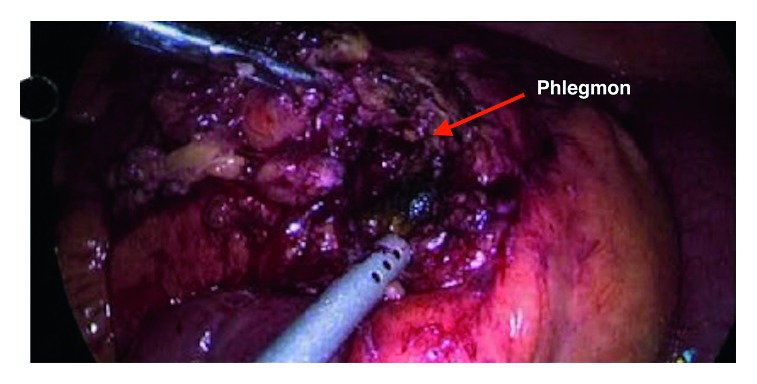


**Figure 3:**
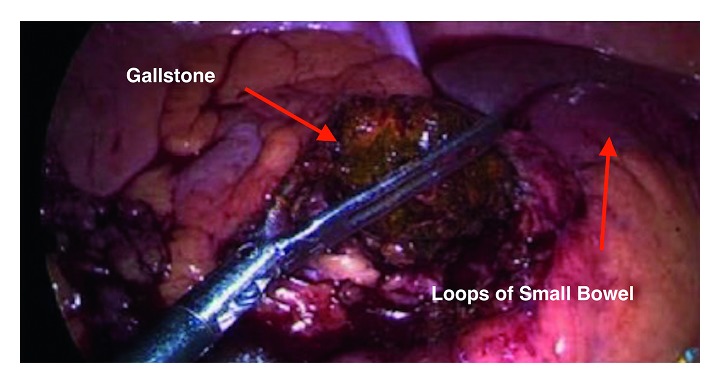


The patient underwent laparoscopy in view of the CT scan findings. Laparoscopy identified a large phlegmon compressing on the distal ileum causing obstruction with distended proximal and collapsed distal small bowel loops (Figure 2). Dissection of the phlegmon revealed a small abscess cavity and the unretrieved gallstone at its center (Figure 3). The abscess was drained and the gallstone retrieved (Figure 4), which resulted in a resolution of the small bowel obstruction. The patient made a gradual recovery and was subsequently discharged on postoperative day 4, at which point he was tolerating solid diet with return of normal gastrointestinal function.

## 3. Discussion

Laparoscopic cholecystectomy has now become the gold standard in treating symptomatic gallstones since its introduction in 1987. Spillage of gallstones during LC is not uncommon [5–7]. It can happen during various stages of the operation such as during dissection of the hepatocystic triangle, dissection of the gallbladder off the liver bed, and retrieval of the gallbladder specimen. However, complication from spilled gallstones is uncommon [11, 12]. They include intraperitoneal abscesses, small bowel obstruction most commonly from intraperitoneal adhesions, and small bowel perforation [13–17]. Small bowel obstruction from spilled gallstones is commonly caused by adhesions that developed as a result of the inflammatory process instigated by gallstones [18–21]. Mechanical small bowel obstruction from a free intraperitoneal gallstone as a result of direct extrinsic compression is extremely rare. To date, we are aware of only one article that mentions extrinsic compression explicitly as a cause of small bowel obstruction [16].

The adverse events, in general, from unretrieved gallstones following LC are uncommon as reported by Manukyan et al. [22]. This was one of the biggest studies, which reported on 580 LCs with a median follow-up of 121 months, one of the longest follows in the literature. They report 22 cases of spilled gallstones with no adverse events. Their optimistic results support the common perception that spilled gallstones rarely cause complications. However, this idea has been challenged by recent reviews, which report complications from unretrieved gallstones ranging from 0.04% to 19% [10].

Jabbari Nooghabi et al. recently reviewed 10 large case series reporting on postoperative complications from spilled gallstones during LC [10]. The study encompassed more than 50,000 patients between 1991 and 2015, reporting more than 250 cases of postoperative complications from spilled gallstones. The study also highlights the fact that most unretrieved gallstones remained clinically asymptomatic and the most common complication from the spilled gallstones is intra-abdominal abscesses [23, 24]. Most of the articles in the literature are case reports [23, 25–38].

The management of spilled gallstones is varied. Known literature suggests that every attempt should be made to retrieve the spilled gallstones irrespective of low rates of complications from them [10, 14, 39, 40]. Several laparoscopic techniques have been described to aid gallstone retrieval when it is lost in the abdominal cavity. They include using a 30-degree laparoscope, additional ports and tissue retractors to visualize difficult corners, a liver retractor to adequately reflect the liver during exploration and suction removal of gallstones, and use of additional endoscopic bags to retrieve the spilled gallstones [41]. Ideally, prevention of spillage of gallstones during laparoscopic cholecystectomy is preferable [42]. This would entail meticulous dissection of the gallbladder from the liver. In spite of the care taken, sometimes gallstones may be spilled when choledochotomy of the cystic duct is performed for intraoperative cholangiogram. In the event of inadvertent gallbladder perforation, the defect may be closed with a loop ligature (Endoloop) or a grasper to minimize spillage [41]. Conversion to open has been mentioned in the literature for retrieval of spilled gallstones, but it is controversial and not justified in the vast majority of cases for retrieval of spilled calculi [8, 10, 43]. Demirbas et al. in their review, which included 9 studies between 1987 and 2013, recommended against conversion to open, as the morbidity associated with retained gallstones is rare [40].

## 4. Conclusion

Complications from unretrieved gallstones are rare. Small bowel obstruction from extrinsic compression from gallstones is extremely rare. The presentation of such complications is often vague and needs definitive diagnosis before considering operative intervention. Our case report emphasizes the need for detailed investigative modalities such as CT scan to accurately diagnose the nature of complication and the required surgical intervention. Although rare, unretrieved gallstones can cause significant complications as witnessed in our case. Operating surgeons should be vigilant in recognizing the spillage of large gallstones. We henceforth recommend laparoscopic retrieval of all spilled gallstones to the best possible attempt to prevent such complications.

## Figures and Tables

**Figure 4:**